# Cultural Adaptation of Child Discipline Measures for Puerto Rican Mothers: Enhancing the Cultural Sensitivity of Parenting Assessments

**DOI:** 10.3390/children11091058

**Published:** 2024-08-29

**Authors:** Jahaira Capellán, Hugh F. Crean, Susan W. Groth, Maria Quiñones-Cordero, José G. Pérez-Ramos, Hyekyun Rhee

**Affiliations:** 1 Department of Pediatrics, University of Rochester Medical Center, Rochester, NY 14642, USA; 2 School of Nursing, University of Rochester, Rochester, NY 14642, USA; hugh_crean@urmc.rochester.edu (H.F.C.); susan_groth@urmc.rochester.edu (S.W.G.); maria_quinones@urmc.rochester.edu (M.Q.-C.); 3 Department of Public Health Sciences, School of Medicine and Dentistry, University of Rochester, Rochester, NY 14642, USA; jose_perez-ramos@urmc.rochester.edu; 4 School of Nursing, The University of Texas at Austin, Austin, TX 78712, USA; hyekyun.rhee@austin.utexas.edu

**Keywords:** parenting, discipline, preschooler, cultural adaptation, measurement, Puerto Rican, health equity

## Abstract

Background/Objectives: Puerto Ricans (PRs) face significant challenges in accessing essential parenting resources and support due to language barriers and lack of culturally appropriate healthcare services, perpetuating health disparities. Cultural adaptation of psychosocial measurement tools is crucial for promoting health equity and improving health outcomes. This study describes the cultural adaptation of two parenting discipline assessment measures for use with Spanish-speaking PR mothers of 2–4-year-old children. Methods: We used a community-engaged, mixed-methods approach to measurement adaptation that involved independent translations (*n* = 2), back-translations (*n* = 2), and an adaptation committee (*n* = 6, including all translators) who reviewed, appraised, and modified survey versions. We conducted cognitive interviews (*n* = 20) to pretest the semi-finalized Spanish measures and assess mothers’ understanding of survey items. Results: Mothers had a mean age of 28.6 years. Most were married/cohabitating (70%), had a high school diploma or GED (90%), and a household income of less than $40,000 (68%). Indexed children’s mean age was 2.9 years, with most identified by mothers as female (60%). Feedback from the adaptation committee and pretesting participants led to specific changes like rephrasing culturally specific terms and adjusting examples to better fit the daily experiences of PR mothers. Most mothers found the Spanish version of the measures to be clear and culturally relevant. This cultural adaptation process addressed translation inconsistencies and design issues, and better captured culturally relevant discipline practices. Conclusions: Engaging communities in measurement adaptations ensures culturally and linguistically tailored measures that respect participant preferences, strengthen partnerships, and enable interventions to address health disparities, thereby promoting child health.

## 1. Introduction

Promoting equity and inclusion in research and healthcare is essential to understanding diverse populations and ensuring that they benefit equally from advances in science and public health; however, a significant barrier is a lack of research instruments created or validated for use with underrepresented groups. This problem is a particular concern in parenting research, which is limited by an inadequate inclusion of racial/ethnic minoritized groups in measurement development and validation studies [1]. The paucity of parenting research in non-English speaking communities hampers our ability to (1) assess the applicability and function of parenting constructs across cultures, (2) accurately comprehend these constructs and their effects, and (3) create meaningful and effective interventions for these populations when needed. 

Parenting practices are behaviors parents use to achieve the critical goals of ensuring their children’s socialization, internalization of cultural values, and safety and health [2]. Discipline is a parenting practice utilized to teach/train children to behave in an acceptable manner, reinforce positive behavior, and foster children’s well-being [3]. Parenting practices, such as child discipline, help to mitigate the impact of social determinants of health, especially poverty, on childhood outcomes [4,5]. For example, parental warmth (e.g., affection and nurturance) has been associated with a lower likelihood of children presenting with a psychiatric disorder (e.g., depression or disruptive behavior disorder), even when a parent uses coercive discipline practices (e.g., physical or verbal punishment) [6]. Effective parenting practices (e.g., communicating expectations and modeling/rewarding good behavior [7]) are important, given that children who are raised in poverty experience a greater risk of health and social problems that can pose a serious threat to their well-being and success later in life [8,9]. 

For many Latinxs, the effects of poverty are often worsened by migration and acculturation stressors, which can impact how they parent and adapt to living in the United States (U.S.) [10]. Additionally, persistent language barriers make it difficult for Latinxs to access essential parenting interventions, resources, and support [11,12]. These challenges are particularly concerning for Puerto Ricans (PRs), who are underrepresented in the parenting literature, despite being the second largest Latinx subgroup, comprising 9% of this population across the 50 U.S. states and the District of Columbia (hereafter U.S.), with their numbers continuing to grow substantially [13]. For instance, in Monroe County, New York, 69% of Latinxs are of PR descent [14], and experience higher rates of poverty. Specifically, 32% of all Latinxs and 40% of Latinx children in Monroe County live in poverty compared with 21% and 28% nationwide, respectively [15]. County-level data from the American Community Survey show that 53% of Latinxs over the age of five report speaking Spanish at home, and 41% indicate that they speak English less than “very well” [16]. These challenges are further compounded by a shortage of Latinx healthcare providers (HCPs) and a lack of culturally appropriate healthcare services and measurement instruments to evaluate and support parenting.

The lack of culturally and linguistically appropriate parenting services not only highlights the barriers this population faces, but also points to the larger systemic issue of underrepresentation in the parenting literature. Nevertheless, the existing literature reveals that Latinx, including PR, parenting practices are guided by the core cultural value of respeto (Respect) [17,18,19]. This value places considerable importance on children being obedient and behaving in an acceptable manner. PR mothers typically bear the primary responsibility for disciplining their children and ensuring that they are behaving appropriately, while fathers are generally expected to enforce the rules [20]. Research has shown that island PR mothers recommend the use of verbal communication, corporal punishment, and removal of privileges most frequently to increase their young children’s compliance and deal with disobedience [21]. Moreover, a study of first-generation Dominican and PR mothers of 2–6-year-olds showed that these mothers used praise and physical affection more often than harsh (e.g., corporal punishment), inconsistent, punitive (e.g., taking away privileges without explanation) parenting practices [22]. It is important to note that given Puerto Rico’s status as a territory of the U.S., PR mothers tend to be more acculturated than other Latinx ethnic groups [23]. Despite this, research indicates that PR mothers living in the U.S. and with extensive exposure to the American culture are more similar to island PR mothers in their parenting than to White mothers [18].

The potential protective role of effective parenting practices in mitigating children’s exposure to adverse social determinants of health underscores the need for a deeper understanding of PR parenting practices. PR mothers, like all parents, can benefit from guidance on effective disciplinary approaches [24]. Despite existing guidelines that emphasize the importance of culturally competent care [7], our knowledge of effective disciplinary methods for PR parents is limited, challenging HCPs who seek to recommend culturally appropriate practices. 

Given the need to better understand PR parents’ discipline practices, we adapted two parenting measures for use with this population. This study addresses the methodological process, challenges, and conclusions drawn from the cultural adaptation of these measures, with insights provided by a sample of Spanish-speaking PR mothers. Cultural adaptation is the process of modifying a measure developed for one culture and language to fit another [25]. It involves systematic, empirical approaches to assess and ensure that the constructs appraised are both relevant and valid for the population of interest [25]. Collecting data in the preferred language and dialect of Spanish-speaking PR mothers is crucial to understanding their culture and discipline practices. Without such an understanding, linguistic and cultural barriers have more impact, hindering research on health disparities and impeding health improvements in this population [26].

## 2. Materials and Methods

### 2.1. Study Design

**Figure 1:**
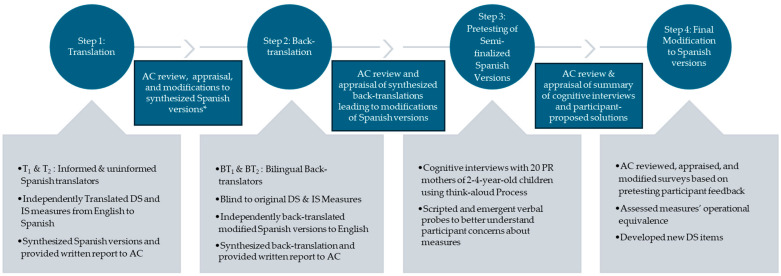
Process of cultural adaptation of the discipline survey (DS) and immediate situation (IS) measurement instruments. Note: T_1_: Translator one; T_2_: Translator two; DS: Discipline Survey; IS: Immediate Situation; BT_1_: Back-translator one; BT_2_: Back-translator two; AC: Adaptation Committee; * Back-translators not included as part of AC at this stage.

This study is part of a larger mixed-methods study conducted between 2016 and 2020. We followed a modified process of cultural adaptation to adapt two parenting discipline measures to the PR culture and language (see Figure 1) [27]. This process involved (1) translation of the measurement instruments from English to Spanish, (2) back-translation of the synthesized Spanish versions into English, (3) use of cognitive interviews with PR mothers to pretest the semi-finalized Spanish versions, and (4) an Adaptation Committee (AC; composed of translators, back-translators, and two mental health experts; all bilingual Spanish speakers) that reviewed and appraised all translations, back-translations, and written reports leading to modifications of the Spanish versions of the measures. Throughout the adaptation process, we sought to maintain the following equivalences: (1) semantic to ensure that the words used in the original and adapted measures relayed the same messages or meanings; (2) idiomatic to develop comparable expressions for colloquial language, which are often more difficult to translate; (3) experiential to replace poorly fitted questionnaire items with similar items that more closely capture the experiences of PRs; and (4) conceptual to modify words that may have different conceptual meanings in the PR culture [27]. Synthesis and decision-making processes were documented in detail. Most AC discussions were audio-recorded. 

### 2.2. Measures

#### 2.2.1. Discipline Survey (DS)

The DS measures parents’ reactive disciplinary practices (behaviors used to reduce or eliminate children’s misbehaviors) and the modes in which these practices are employed [28]. This measurement instrument is composed of 32 items assessing 10 types of reactive discipline and 6 modes of administration with Cronbach’s alpha values as reported by Socolar and colleagues (2004) [28]. The discipline practices are measured by five subscales: Modeling Behavior (e.g., show child how to behave; 3 items; *α* = 0.82), Verbal Communication (e.g., talk about problem; 3 items; *α* = 0.76), Ignoring Behavior (e.g., withdraw attention; 3 items; *α* = 0.64), Monitoring (e.g., let child know you are watching; 2 items; *α* = 0.74), and Corporal Punishment (e.g., spank; 2 items; *α* = 0.78) in addition to five single items: Distract, Natural Consequences, Time Out, Reward (when behaving well), and Remove Privileges [28]. The modes of administration are measured by four subscales: Positive Parental Demeanor (e.g., calm; 3 items; *α* = 0.56), Negative Parental Demeanor (e.g., angry; 3 items; *α* = 0.56), Consistency (e.g., child knew what to expect; 3 items; *α* = 0.57), and Follow-Through (e.g., do what you said you would; 3 items; *α* = 0.71) in addition to two single items: Belittling Demeanor (i.e., embarrassing child) and Stern Demeanor (i.e., firm and serious) [29,30]. All responses are assessed using a 6-point Likert-type scale ranging from 1 (never) to 6 (always). They concentrate on parental behaviors used to address the target child’s misbehavior in the last 3 months. Scores for each subscale are based on the mean of item responses for that subscale [28]. The DS also includes seven sub-questions to evaluate (1) the frequency of child misbehavior, (2) the amount of time that parents spoke, spanked, and withdrew privileges from their child, and (3) how the participating parent was disciplined as a child.

#### 2.2.2. Immediate Situation (IS)

The IS measurement instrument evaluates whether specific circumstances/situations affect parents’ choice of discipline [29]. It consists of 11 items comprising 4 subscales: Type of Misbehavior (e.g., what child did to misbehave; 2 items; α = 0.77), Parent–Child Interaction (e.g., how child reacted when disciplined; 4 items; α = 0.71), Location (e.g., whether in public; 2 items; α = 0.71), and Temporary Stressors (e.g., parent having a bad day; 3 items; α = 0.74) [29]. Please note that Cronbach’s alpha values are as reported by Socolar and colleagues (2005). Responses are recorded on a 6-point Likert scale ranging from 1 (not at all) to 6 (very much). Subscale scores are determined by calculating the mean item score. IS subscales are typically related to most of the DS subscales in expected patterns [29]. 

A benefit of the DS and IS measures is the inclusion of modes of reactive discipline administration and the immediate situations influencing parents’ approach to discipline [28]. To date, neither instrument has been translated nor adapted from their English versions. We took a first step by adapting these measures into culturally specific Spanish versions. 

### 2.3. Cultural Adaptation Process

#### 2.3.1. Translation

The DS and IS measures were translated from English to Spanish independently by two bilingual HCPs (T_1_ and T_2_) whose primary language was Spanish. They demonstrated their language proficiency by effectively delivering patient care and education in both languages and accurately documenting care in English. T_1,_ the principal investigator (PI), had formal training as a Spanish interpreter and knowledge of the discipline concept as described in the literature. T_1_ is of Dominican descent and has worked extensively with the PR population in clinical practice. T_2_ is a PR mother of an 11-year-old child and was naïve to the underlying concepts of the measures. After completion of the translations, the two translators met to discuss any differences/concerns, reached a consensus, and synthesized the translations into preliminary Spanish versions of the measures. 

#### 2.3.2. Back-Translation

To ensure that the translated Spanish versions accurately reflected the original English versions, two additional translators (BT_1_ and BT_2_) followed a similar process to independently back-translate the synthesized Spanish versions into English [27]. Both back-translators were bilingual PR health professionals proficient in English and Spanish. They were masked to the original English measures. Once both back-translations were completed, these translators met with the PI (T_1_) to synthesize the back-translations of the DS and IS. During this process, any errors and/or discrepancies in interpretations of back-translated items were examined and discussed until consensus was achieved among the three translators. 

#### 2.3.3. Adaptation Committee (AC) Appraisal and Modifications of Translations

After the back-translations were completed, the AC met to review and evaluate any inconsistencies between the language of the original and the synthesized translations. The committee consisted of the two translators, the two back-translators, and two bilingual experts. One expert was a Panamanian mental health provider who was a certified Spanish translator and worked closely with the PR population; the other was a PR with a master’s degree in Marriage and Family Therapy. All AC members read and reviewed all three DS and IS versions (original, synthesized translations, and synthesized back-translations). Discrepancies across the versions and/or concerns about linguistic clarity and cultural sensitivity of the measures were discussed and addressed until consensus was reached, creating semi-finalized Spanish versions of the DS and IS to use for pretesting. 

#### 2.3.4. Pretesting of the Adapted Instruments 

To safeguard against the inclusion of items that could be misleading or lack equivalence, the committee-approved Spanish translations were pretested with 20 PR mothers of 2–4-year-old children [27,31]. Mothers were recruited from the local community through snowball sampling, word of mouth, and placing study flyers at local churches and businesses (e.g., restaurants and hair salons) serving this population. After obtaining consent, the PI interviewed participants in Spanish for approximately 60 min at their homes. Interviews were conducted until no additional issues or insights were identified. Interviews were audio-recorded and detailed notes were taken and reviewed after each interview. Participants were compensated with USD 25 for their time. 

##### Cognitive Interviewing Using the Think-Aloud Process

Cognitive interviewing is designed to gather insights into participants’ interpretation of questionnaire items and response categories by requesting them to verbalize their thoughts as they examine and answer items on the questionnaires [32]. To evaluate which survey items participants struggled to understand, we used the think-aloud technique of asking mothers to read questions aloud and talk through their decision-making process [33,34]. Prior to initiating these interviews, participants were trained in the process of ‘thinking aloud’ by using two practice questions from the Cultural Socialization of Latino Children measure [35]. These questions were (1) “I believe that children should obey no matter what”, and (2) “It is not acceptable for children to interrupt adult conversations” (both answered from 1 = Strongly Disagree to 5 = Strongly Agree). If participants struggled during the think-aloud sessions, the following prompts were used: (1) “Tell me what you are thinking” or (2) “What are you thinking about right now?” [32]. 

##### Probes Used during Pretesting

Verbal probes were used to help clarify any participant’s difficulty with stem-question recall, comprehension of questionnaire items, and/or response category options. These probes helped the investigator to understand the participants’ decision-making process [27,33,36]. Ten scripted probes were available to be asked at the end of the think-aloud sessions (see Table 1). Emergent probes were developed during survey administration and used in later think-aloud sessions to capture any concerns raised by research participants [37]. Examples of emergent probes included (1) What does the phrase ‘llevarse bien en el mundo’ (get along in the world) mean to you? and (2) What does the word ‘recompensa’ (reward) suggest? (see Table 1). 

##### Documentation and Data Analysis 

Memos were written as the study progressed and included the PI’s thoughts and insights about the developing codes and themes [39]. Continuous data analysis was used throughout the process. The audio-recorded cognitive interviews were transcribed verbatim and translated into English by the PI. The collected memos and de-identified participant data were organized and analyzed question-by-question, using thematic qualitative analysis [40]. Specifically, open coding was utilized to identify initial codes. They were then grouped into categories to create themes. The developed themes were reviewed and discussed with all AC members until a consensus was reached. These themes were used to (1) evaluate whether the measures appeared to effectively assess the intended discipline concepts and (2) develop additional items related to the Positive and Negative Demeanor concepts to help improve the internal consistency of these respective subscales [41]. In addition, deductive coding was conducted using the ‘translation, cultural adaptation, generic problem’ (TCG) system [42], described below. The TCG system helped to inform the development and identification of translation-related themes used to further adapt the Spanish versions of the DS and IS. 

Translational problems represent linguistic issues caused by errors in the translation methodology [42], such as mistranslation of a specific concept or errors in grammar. For example, if a participant struggled to comprehend a certain term, they were asked if there was a word to better capture the targeted meaning. In reporting this type of semantic change, we described the following key components: (1) the Spanish word of concern; (2) a verbatim translation of the word’s semantic meaning, its significance to the PR culture, and how it is utilized; (3) reasons respondents interpreted a specified word in a particular manner; (4) suggestions for use of another word; (5) translation of a new word into English; and (6) explanation behind the use of the new word [37]. 

Cultural adaptation problems are indicative of survey items that fail to operate in the same manner across populations [42]. This can occur because of cultural differences in worldviews. To address this challenge and ensure a better fit of the DS and IS measures to the PR population, the objectives of survey questions were clarified and specific items with similar meanings were developed [34]. 

Lastly, generic problems represent issues/defects with the original DS and IS items that transcended culture and were carried over to the Spanish translation [42]. Specifically, issues that interfered with a participant’s ability to answer questionnaire items were recorded. Prior to assigning observations to a specified TCG category, the problems elicited by the participants were summarized across interviews to create related themes [43]. Based on these themes, written suggestions to modify specific items were provided to the AC. 

#### 2.3.5. Adaptation Committee (AC) Reappraisal and Modifications

The AC met to discuss and evaluate concerns elicited from the pretesting participants. To facilitate these deliberations, the PI transcribed and summarized recorded encounters and participant-proposed resolutions. All six AC members needed to concur before any survey changes were made. Based on feedback from the pretesting participants and what is known from the literature, the committee assessed the measures’ operational equivalence by determining the adequacy of their format, layout, and application for administration in the PR population [44]. After the AC reached a consensus, the appropriate semantic and operational changes were made to create the adapted Discipline Survey—Spanish (DS-S; see Table A1) and Immediate Situation–Spanish (IS-S; See Table A2) measures. 

### 2.4. Ethical Considerations

Because the owner of the DS and IS measurement tools was deceased, permission to culturally adapt them was obtained from the owner’s next of kin. This study was approved by the University of Rochester Research Subjects Review Board (Study number: RSRB00059732). Written consent was obtained from participating mothers. 

## 3. Results

### 3.1. Report on the Synthesized Translation 

The following results pertain to the translations of both the DS and IS measures. The translations of each measure by T_1_ and T_2_ were relatively similar and generally conveyed the meaning of the original questionnaire items. Metaphrase (word-for-word translation), along with sentence structure arrangements, were used in both translations. A total of 41 differences were noted between the completed translations. Many reflected variations in conjugation and the use of synonyms for the same words. To resolve these discrepancies, the translators deliberated until they reached a consensus. As a sign of respect for future participants, they decided to use the more formal pronoun ‘usted,’ when conjugating verbs that referred to the pronoun ‘you.’ They also chose Spanish words that were more commonly understood by PRs. 

In ensuring semantic equivalence, a few mutually agreed-upon decisions were made by the AC. For example, one of the DS survey items (what would you say was the main method used to discipline you when you were a child?), required further explanation to ensure semantic equivalence. Spanish definitions for the parent punishment practices of time-out (time spent alone) and grounding (time that you could not go out) were used because exact words for these concepts do not exist in the Spanish language. While the Spanish word ‘castigo’ refers to punishment, it does not specify the actual (or specific) type of punishment. 

Maintaining experiential equivalence was a concern for survey items related to spanking (e.g., did you spank him/her?). The experience of PR mothers in disciplining their children through spanking/corporal punishment includes more than slightly hitting on the buttocks (nalgadas). For example, the child may be hit on the mouth (tapaboca) if he/she says a bad word or on the head (cocotazo) if he/she is being hardheaded [24]; thus, the more comprehensive words for ‘hitting’ (pegar and dar) were used instead of spanking. Lastly, there was a need to achieve idiomatic equivalence for the item ‘did you slap or pop his/her hand?’ Since the colloquial term ‘pop’ is not used in the Spanish language, both translators used the phrase ‘did you hit his/her hands’ (le dio en sus manos) to convey the essence of this item.

### 3.2. Report of the Synthesized Back-Translation

The two back-translations were similar and maintained the semantic equivalence of the original questionnaire items. A total of 60 differences were noted between the two back-translations, many of which were related to differences in sentence structure or the use of synonyms to translate the same concepts/words. Ultimately, the words that were more like the original English version were chosen. If the back-translated word or item was not like the original English version, other Spanish words that might better target the item/word’s essence were explored to better achieve semantic equivalence. 

There were some instances in which the back-translated words did not resemble the original measures or needed further modifications. For example, this was noted in the response options for the DS (never, rarely, sometimes, often, almost always, always) and IS (very much, quite a bit, some, not really, not much, not at all). In terms of the DS responses, ‘muchas veces’ was translated by both back-translators to ‘many times’. While ‘many times’ is defined as ‘often’ [45], it was perceived to have the same or a higher degree of frequency than ‘almost always’. To maintain semantic equivalence, ‘muchas veces’ was changed to ‘frecuentemente’, which directly translates to ‘often’ [46] (see Table 2).

The IS response options proved to be problematic and appeared to be a limitation of the original measure. Although the intention of the response scale was presumably to decrease in frequency from left to right, the last three response choices did not appear to be in the correct order despite being accurately translated. ‘Not much’ was thought to imply that behaviors could sometimes happen in a small amount and should, thus, come before ‘not really.’ In defining these two words, ‘not much’ is described as “a small amount of (something)” [47], while ‘not really’ is construed as an idiom “used to say ‘no’ in a way that is not very forceful or definite” [48]. The first three categories in the IS response set also required modification to achieve semantic equivalence. The final synthesized back-translated response options read as follows: very much, often, sometimes, not much, not really, never (see Table 2). 

Finally, for DS-S item #4, a concern with the word ‘discipline’ (disciplina) emerged (see Table 3). While this word is used in the PR population, it is not commonly used by the average PR. To further clarify its meaning, the word ‘corregir’ (‘to correct’) was added to this question. 

### 3.3. Adaptation Committee (AC) Appraisal and Modifications of Translations

The AC’s review resulted in several items requiring modifications to the synthesized Spanish version of the surveys prior to pretesting. These adjustments were related to the DS-S items associated with modeling (#8), spanking (#11), and lack of follow-through (#24). Further revisions were also needed for the IS-S instructions and response options (see Table 2). These modifications maintained the item-semantic equivalence and were approved by all AC members. 

There was concern that, as written, DS-S item #8 could be perceived as offensive to some mothers. It was thus adjusted to ‘did you demonstrate to NAME how to behave in the correct way when he/she misbehaved so that he/she could imitate your behavior?’ (see Table 2). To diminish the potential perceived harshness of the word ‘pegar’ (to hit), item #11 was modified by adding the word ‘nalgadas’ (‘hitting the butt’) to the list of presented examples (see Table 3). Finally, DS-S item #24 needed modifications as the AC favored the phrase ‘se dio por vencida’ for a more accurate representation of ‘give in.’ This decision was validated by verifying that ‘give in’ translates to ‘darse por vencido’ from English to Spanish [49].

Regarding the IS-S measure, the word ‘situations’ (situaciones) was added to the instructions to clarify that when using ‘each of the following,’ it would refer to different specific situations. Lastly, the IS-S response options were further adapted to ensure that the scale was indeed decreasing from left to right. With this goal in mind, the word ‘a menudo’ (often) was replaced by its synonym ‘frecuentemente’, ‘un poco’ (a little) was used to substitute for ‘no mucho’ (not much), and ‘casi nunca’ (almost never) took the place of ‘no realmente’ (not really). Thus, the final IS-S response options are now as follows: very much, often, sometimes, a little, almost never, and never (see Table 2). 

### 3.4. Pretesting and Adaptation Committee (AC) Appraisal and Modifications

The age of the 20 participating mothers ranged from 21 to 40 years (mean = 28.6 years, *SD* = 4.36). Most mothers reported being either married (25%) or cohabitating (45%), having a high school diploma or GED (90%), and a household income of less than $40,000 (68%). The mean age of index children was 2.9 years, with most identified by mothers as female (60%). 

Participant responses during the cognitive interviews largely confirmed, as intended, that the pretested DS-S and IS-S measures assessed PR mothers’ discipline practices, modes of administration, and situations influencing these practices. Participants raised a total of 22 concerns in understanding the questionnaire items. Most concerns were translational problems related to words like: ‘enfado’ (anger), ‘avergonzar’ (embarrass), and ‘ánimos’ (encourage). Although most mothers understood these terms, a small number had difficulty comprehending them, possibly due to unfamiliarity with the vocabulary used or their educational/reading background. We addressed these challenges by exploring other word alternatives with participants. In addition, to better assess and understand these findings and determine the best solution or word alternative, emergent probes were developed for use with subsequent participants. A more significant translational concern was related to the reward–discipline practice. While we found a word for ‘reward’ (recompensa), a few mothers struggled to understand it and suggested that examples be provided; therefore, a few examples of rewards were included for this item (see Table 3). No significant cultural adaptation problems were identified during pretesting.

Three main concerns arose in the generic problems category. They included (1) difficulty interpreting the word NAME in the provided context, (2) challenges in remembering the item stem, which led to problems answering questions accurately, and (3) the length of item #4 caused confusion. To resolve the first generic problem, we added the following in parenthesis: NAME represents the name of your son/daughter (NOMBRE representa el nombre de su hijo/a). The second generic problem was amended by including the stem in each item and italicizing the remaining portion of the question. The third generic problem was addressed by deleting the word ‘like’ and putting the detailed item examples in parenthesis (see Table 3). This solution allowed participants to focus on the main question, using the examples if additional clarification was required. 

Eight themes were identified from the participants’ cognitive interviews and open-ended question responses (see Table 4). These themes were used to develop eight additional DS-S items with the intention of better capturing the specific cultural practices and experiences of PR mothers (see Table 4). Two of these items were about the common discipline strategy of yelling or scolding when a misbehavior occurred, while the other six were about Negative Demeanor (4 items) and Positive Demeanor (2 items). The stem and response options for these additional items were the same as those used in their respective categories. While these additions contributed to cultural relevance, their psychometric properties, as well as those of the adapted instruments, require further study.

Quantitative data from the cognitive interviews further supported the preliminary validity of the measures. PR mothers in this study frequently endorsed the discipline practices and modes of administration included in the DS-S, indicating the relevance of the items. For example, 90% of mothers reported using Verbal Communication and Modeling Behavior often or more frequently (see Table 5). While practices such as Corporal Punishment and Ignoring Behavior were used less often, 75% and 60% of mothers, respectively, reported using these practices. Mothers endorsed all modes of administration except for Belittling Demeanor, with 95% of mothers reporting that they never embarrassed their children. The Cronbach’s alpha for the discipline practices subscales, based on the factor structure of the original measure, ranged from 0.44 to 0.71, while that of the modes of administration ranged from 0.19 to 0.72. It is important to note that these calculations did not account for the newly created items, as they were developed during the cognitive interviewing process, and not all participants responded to these new questions.

Regarding the IS-S, 70–85% of mothers reported that the situations assessed affected the way they disciplined their child at some level, ranging from very much to almost never (see Table 6). These findings indicate that the items are understood and relevant to PR mothers’ lived experiences. The Cronbach’s alpha for the IS-S subscales, based on the factor structure from the original measure, ranged from 0.66 to 0.85.

## 4. Discussion

We adapted the DS and IS tools for use with Spanish-speaking PR mothers, employing an iterative, community-engaged approach. This methodology allowed us to apply a systematic strategy that supported the cultural adaptation of these measures, going beyond simple translation. Our findings underscore the importance of culturally adapting existing measures to consider culture, values, and language. Such adaptation is crucial for research that explores potential protective factors like parenting practices that can mitigate the effects of social determinants of health on childhood development, including parenting practices. 

Several lessons emerged while adapting the DS and IS for Spanish-speaking PR mothers. The first lesson involved difficulties with translations; that is, the need to carefully adjust for (1) word conjugation, (2) pronouns for gender-specific nouns, and (3) sentence syntax or grammatical ordering of subject, verbs, and objects when translating measures into Spanish [50,51]. The use of a dialect and vocabulary that could be readily understood by PRs was also critical. To help address this concern, commonly used words were chosen over direct translation equivalents that were less often used and presumably less understood within the PR population. For example, the word ‘corregir’ was used for discipline in addition to its direct translation ‘disciplinar.’ Furthermore, changes in language often led to the use of more words to communicate linguistically non-equivalent language or expressions [50]. Longer questions can lead to difficulty in understanding and may produce a cumulative fatigue effect. We addressed this concern by breaking down complex sentences into simpler structures and parenthetically adding examples where they were deemed helpful. In addition, colloquial terms were avoided to facilitate future adaptation and psychometric testing of these measures for other Spanish-speaking groups. 

The second lesson confirmed that translation alone is not enough when adapting measures as they may result in items being biased and having differing connotations [50]. An example in this study was the use of words that sounded similar but meant different things like ‘cedió’ (to cease) and *‘se dio’* (to give). An important strength of our work was engaging an AC of bilingual Latina women in translation, back-translation, and pretesting. This enriched the discussion of mothers’ parenting practices and safeguarded against biased and inappropriate items [27].

Through the adaptation process, several translational and generic problems were identified and addressed. While generic problems (e.g., problems with the original DS and IS) constituted only a few of these concerns, one was a significant limitation to both the existing and adapted measures. It required modification of the IS Likert response options to ensure that they decreased in frequency from left to right, with more equidistant spacing between choices. The absence of cultural adaptation problems during the pretesting stage can be attributed to our focus on maintaining experiential equivalence during the translation and back-translation steps. During this phase, attention was given not only to linguistic accuracy, but also to ensuring that the survey items reflected the discipline experiences of PR mothers. 

The third lesson reinforced the importance of considering both culture and language when adapting measures for other populations, as items may behave differently across cultures [52]. Accounting for the cultures of the source and target populations is critical in informing research to ensure that the concepts being measured are relevant and have equivalent meanings in both populations. In this study, our focus was on assessing whether the constructs measured by the DS and IS remained relevant and operated similarly in the PR sample, consistent with their functioning in the Black and White samples originally used to validate them. Attention to cultural context and language allowed us to identify and address discrepancies, ensuring that the adapted measures accurately reflected the constructs they were intended to assess. 

Ideally, an adaptation should maintain the semantic equivalence of the original measure as much as possible. Yet, to effectively do so, items whose literal translations result in cultural bias should be modified [51]. In adapting the DS measure, we encountered challenges due to lexical gaps: instances where a word or concept in English lacked a direct equivalent in Spanish [53,54]. For example, we faced difficulty translating the disciplinary practices of Reward, Time Out, and Corporal Punishment because the literal translations did not fully capture important cultural nuances. Translating these practices required adjustments to better convey the intended meaning within the PR cultural context. 

Although we found a word for ‘reward’ (recompensa), we had to provide examples of the discipline strategy to make this item more culturally equivalent. The examples we originally listed (i.e., purchase of an item or activity) failed to include any non-monetary forms of this practice, which was needed for this cultural group. This idea is supported by a qualitative study of treatment acceptability of parenting practices, which found that most Latina mothers, including PRs, opposed the use of material rewards, as they did not perceive it to be a feasible practice, given their limited financial circumstances [55]. In retrospect, praising the child’s good behavior would have been an important example of a reward to include. Verbal praise is often used by PR parents as a reward strategy in parenting [22,23]. This finding suggests that, in future studies, it would be valuable to examine how PRs use different types of rewards for positive reinforcement [55]. 

The item related to the discipline practice of Time Out also required modification because a specific Spanish term could not be identified, despite mothers’ acknowledging the use of this strategy. It is likely that Time Out is used less often in the PR culture. For example, research has demonstrated that PR parents who had not been acculturated to the American culture and were living in Puerto Rico were not willing to adopt the use of a time-out room as a disciplinary practice [56]. Even when these parents were able to view their 4–6-year-old child through a one-way mirror, they felt distressed about using a time-out room. In addition, Latin Americans born in the U.S. have been shown to perceive Time Out as more effective than their foreign-born counterparts [57]. Thus, for PR mothers living in the U.S., acculturation may contribute to their awareness, comprehension, and perceptions of the suitability of this practice [55]. 

The items related to corporal punishment required modifications as well to attempt to minimize item bias (the risk of a response based on social desirability), and the chances that PR mothers would be less forthcoming about the use of this practice. Research shows that PRs perceive spanking to be an acceptable discipline practice to instill the value of *respect* and ensure that children obey and behave properly [21,58,59]. Nonetheless, PR mothers may fear that the surrounding American culture would misconstrue spanking as abusive. The potential legal ramifications of using corporal punishment may leave parents feeling disempowered when raising their children [55]; thus, we felt it was essential that the translation of spanking encompassed the specific forms of this practice that mothers engaged in, while avoiding terms that may be perceived as too severe or non-normative. These translational concerns reinforced the importance of considering culture when translating terms and words [19]. 

It is important to note that all participants indicated that the items assessing discipline practices, modes of administration, and situations included in the adapted DS and IS were pertinent to PR mothers’ discipline encounters. Because many PR mothers reported disciplining children by yelling and scolding, we added two new questions related to these specific practices. Another study of PR mothers, in contrast, found that 55% perceived verbal reprimands (e.g., screaming, negative scolding, insults, etc.) to be an inappropriate discipline strategy to deal with disobedience and non-compliance in children under 6 years of age [21]. However, their definition of a verbal reprimand was broader and included practices such as insults, swearing, and belittling, which may be perceived to be abusive and demeaning. 

In this study, we adapted the DS and IS measurement instruments for use with Spanish-speaking PR mothers through a community-engaged adaptation process. While we calculated and reported Cronbach’s alpha using the original measures’ factor structure, caution is advised in interpretation due to our small sample size. Specifically, research has shown that estimates of Cronbach’s alpha require a sample size of 30 or more to achieve stability and accuracy [60]. Additionally, we did not have enough responses to the newly created DS-S items to conduct a factor analysis and add these items to Cronbach’s alpha calculations. There is a possibility that the factor structure for the PR population may differ from that of the original measure. Despite this, the primary objective of achieving linguistic and cultural relevance was met, although with the understanding that further psychometric validation is necessary. Future studies with larger sample sizes are needed to determine the factor structure of these measures, assess the measures’ psychometric properties, including validity and reliability, and further strengthen their overall robustness. This would help to expand our understanding of PR discipline practices, enabling researchers and HCPs to develop and implement effective, culturally attuned recommendations. In doing so, they could potentially cultivate a more professional and trusting partnership with PR parents—promoting successful socialization and minimizing developmental and behavioral challenges among PR children [61,62].

### 4.1. Limitations

A limitation of this study is that despite the AC being composed of all bilingual Latinas, the cultural/ethnic background of two of the six members was not PR. This limitation was likely offset by their close work with the PR population in clinical practice, as well as our thorough pretesting of the DS-S and IS-S measures with PR mothers. Cognitive interviews provided direct insights into the PR culture, aiding in the cultural relevance and comprehensiveness of the adapted measures. This approach helped address cultural nuances that may have been overlooked by the AC. Future studies should consider the use of a standardized translation and adaptation review form to ensure that all AC members are systematically and consistently addressing adaptation concerns. 

Another limitation of this study is that we were unable to conduct factor analyses on the fully modified or adapted measures with this small sample size. This prevented us from determining the factor structure and assessing the internal stability of the respective subscales. Consequently, the reliability and validity of the newly adapted items could not be thoroughly evaluated. This limitation highlights the need for future research to include larger PR samples to facilitate factor analysis and ensure the psychometric robustness of the adapted measures.

Lastly, this study only included PR mothers living in the U.S., so its applicability to fathers and parents living on the Island of Puerto Rico is unknown. As in all cultures, fathers and other important caretakers serve critical roles in children’s care and development. Future studies should aim to include these groups to improve the generalizability of the findings. 

### 4.2. Implications for Clinical Practice and Public Health

Given the need for HCPs to provide culturally competent care and disciplinary guidance for Spanish-speaking PR families, our adaptation of the DS and IS measures provides potentially useful tools to develop a culturally appropriate understanding of PR disciplinary practices. This knowledge can help to strengthen HCP–parent partnerships to promote child health and assist clinician researchers in creating culturally and linguistically tailored interventions that address health disparities and foster community trust in public health services. 

## 5. Conclusions

This study provides guidance on navigating the complexity of translating and adapting existing research instruments. We have demonstrated in detail how cultural adaptation methodology, including translation, back-translation, expert committee review, and pretesting of the measures, was applied to adapt two parenting measures. Spanish versions of the DS and IS were developed and demonstrated relevance with the target population of PR mothers of 2–4-year-olds. The adaptation resulted in the addition of six new items for the demeanor subscales and two more discipline practices applicable to this population. Clarification of response scales for both measurement tools was also achieved. Our linguistic and cultural translation of the DS and IS may help expand the study of parental discipline practices involving PR populations. Such research also contributes to health and language equity for the often-underrepresented Spanish-speaking PR population, offering measures that are more easily understood and culturally relevant in assessing key practices used in disciplining their children. Effective measurement of these practices can help to increase providers’ knowledge of the types and extent of discipline practices used, and ultimately lead to designing and tailoring culturally attuned parenting interventions for this population. 

## Figures and Tables

**Table 1:** Scripted and emergent probes used in think-aloud sessions (TASs).

Scripted Probes (Asked after TASs)	Emergent Probes (Asked during TASs)
Overall, do you think we captured the ways that you correct your child’s behavior?	What does the phrase “llevarse bien en el mundo” (get along in the world) mean to you?
Did the words, terms, and discipline practices in this survey sound appropriate to your PR culture of origin?	What does the word “recompensa” (reward) suggest? Is there a better way to say this in Spanish? Would providing examples help to clarify this question?
Do people where you come from speak in the same way used in this survey?	How do you say “sticker” in Spanish?
Are there ways of disciplining your child that are common in your PR culture that were not described here?	Does the word “requirió” (require) make sense to you? If not, what word would you use in this context?
Were there any practices or terms that did not seem to be appropriate [38]?	Do you understand the phrase “estuviera solo/a por un tiempo breve” (to be alone for a brief time) to mean time out?
Did these questions make sense to you?	Is the word “papao” (slightly hitting on hands) a form of spanking and does it help to clarify what is meant by the word “pegar” (to hit)?
Did you see the answer you would choose?	Does the phrase “dejar de prestar atención” (stop paying attention) make sense to you?
Were there any words that seemed funny or did not make sense?	Is “enfogonó/se molestó” a better translation for “got upset” than “se enfadó”?
“Was there anything that caused you concern” [38] (p. 10)?	Do you understand the meaning of “abochornar/avergonzar” (embarrass)?
Were there any questions that made you feel uncomfortable?	Is the word “motivaran” a better translation for “motivate” than “dar ánimo”?

**Table 2:** Adaptation process: discipline survey (DS) and immediate situation (IS) items requiring further modifications.

Item #	Original	ST	SBT	SV: Post AC	Pretesting	Final—English
DSR	Never Rarely Sometimes Often Almost Always Always	Nunca Raramente A veces Muchas veces Casi Siempre Siempre	Never Rarely Sometimes Many times Almost Always Always	Nunca Raramente A veces Frecuentemente Casi Siempre Siempre (AWP-SBT)	No change	Never Rarely Sometimes Often Almost always Always
8	Did you demonstrate good behavior…when he/she misbehaved…	Demostró usted buen comportamiento… cuando él/ella se portó mal…	Did you demonstrate proper behavior …when he/she misbehaved…	Le demostró…cómo comportarse de manera correcta cuando él/ella se portó mal…	No change	Did you demonstrate …how to behave in the correct way when he/she misbehaved…
24	Did you back down, give in, and not discipline, if he/she got upset?	Se echó para atrás, cedió, y no disciplino, si él/ella se enfadó?	Did you stand back, ceased, and did not discipline, if he/she got angry?	Se echó para atrás, se dio por vencida, y no disciplinó, si él/ella se enfadó?	…se ‘enfogonó’ o molestó?	…did you back out, give up, and did not discipline if he/she got angry/ upset?
ISI	How much would you say each of the following affected the way you disciplined …?	¿Cuánto diría usted que cada una de las siguientes ha afectado la manera en que usted a disciplinado…?	How much would you say that each of the following has affected the way you disciplined…?	¿Cuánto diría usted que cada una de las siguientes situaciones ha afectado la manera en que usted a disciplinado…	No change	How much would you say that each of the following situations have affected the way you disciplined…?
ISR	Very much Quite a bit Some Not really Not much Not at all	Mucho Bastante Un poco No realmente No mucho Para nada	Very much Often Sometimes Not much Not really Never	Muchísimo Frecuentemente Algunas veces Un Poco Casi nunca Nunca	No change	Very much Often Sometimes A little Almost never Never

Note. #: Number. ST: Synthesized Translation; SBT: Synthesized Back-translation; SV: Spanish Version; AC: Adaptation Committee; AWP: Agreed with Post; DSR: Discipline Survey Response; ISI: Immediate Situation Instructions; ISR: Immediate Situation Response.

**Table 3:** Adaptation process: discipline survey—Spanish (DS-S) items requiring examples for further clarification.

I #	Original	ST	SBT	SV: Post BT	SV: Post ACR	Pretesting	Final—English
4	Rather than …disciplining …did you let him/her handle the consequences …—like…	En vez de disciplinar… dejó que él/ella manejara las consecuencias… —como…	Instead of disciplining/ correcting… did you let him/her deal with the consequences… —like…	En vez de disciplinar/ corregir… dejó que él/ella se enfrentara a las consecuencias … —como…	Agreed	…en vez de disciplinar/ corregir…dejó usted que él/ella se enfrentara a las consecuencias …(p. ej…)	…instead of disciplining/ correcting… did you let him/her deal with the consequences… (e.g., …)
7	Did you give …a reward…	Le dio…una recompensa…	Did you give … a reward…	No change	No change	…le dio a…una recompensa/ premio (p. ej., dulce, estiquer, o ir a su lugar preferido como Sky zone o Chuck E. Cheeses)	…did you give…a reward (e.g., candy, sticker, or go to his/her favorite place like Sky Zone or Chuck E. Cheeses)
11	Did you spank him/her?	Le pego usted a él/ella? (p. ej., tapaboca, cocotazo)	Did you hit him/her?	No change	…le pego usted a él/ella? (p. ej., nalgadas, tapaboca, cocotazo)	No change	…did you hit him/her? (e.g., hitting on the butt, mouth, or head)
FSI	How many times did you usually spank…	¿Cuántas veces por lo general le dio…	Usually how many times did you hit…	No change	No change	Por lo general, ¿cuándo le dio (p. ej., nalgadas o ‘papao’)…	In general, when you hit (e.g., butt or hands)…

Note. I #: item number; FSI: Freestanding Item; ST: Synthesized Translation; SBT: Synthesized Back-translation; SV: Spanish Version; ACR: Adaptation Committee Revision.

**Table 4:** Emerging themes informing development of additional DS-S questions from pretesting.

Discipline Strategy	Demeanor	
	Positive Demeanor (calm and respectful)	Negative Demeanor (angry and hostile)
did you **yell** at him/her? (¿le gritó a él/ella?)	did you **take deep breaths prior to disciplining** your child? (¿respiró profundo varias veces antes de disciplinar/corregir a NOMBRE?)	were you **frustrated**? (¿diría que estaba frustrada?)
did you **scold** him/her? (¿lo/la regañó?)	did you **stop and think prior to disciplining**? (¿paró y pensó cómo iba a responder antes de disciplinar/corregir a NOMBRE?)	were you **embarrassed**? (¿diría usted que se sentía abochornada o avergonzada?)
		were you **trying to scare** NAME? (¿estaba tratando de asustar o darle miedo a NOMBRE?*)*
		did you **react immediately without thinking**? (¿reaccionó inmediatamente sin pensar cuando disciplinó a NOMBRE?)

Note. The words in bold are the emerging themes used to develop the corresponding questions. The stem of discipline strategy questions was “In the last three months when you had a problem with the way NAME behaved…” The stem of demeanor questions was “When you were handling a behavior problem in the last 3 months…” [28].

**Table 5:** Frequencies of disciplinary practices and modes of administration as reported by mothers (N = 20).

**Type of Discipline**	**Never (%)**	**Rarely (%)**	**Sometimes (%)**	**Often (%)**	**Almost Always (%)**	**Always (%)**
Verbal Communication	0	0	10	20	30	40
Modeling Behavior	0	5	5	40	35	15
Monitoring	0	0	10	25	45	20
Corporal Punishment	25	45	15	15	0	0
Ignoring Behavior	40	35	25	0	0	0
Distract ^	15	0	25	15	35	10
Natural Consequences ^	40	15	15	15	15	0
Time Out ^	5	5	30	15	20	25
Remove Privileges ^	10	0	25	20	30	15
Reward ^	10	5	20	10	25	30
**Mode of Administration**	**Never (%)**	**Rarely (%)**	**Sometimes (%)**	**Often (%)**	**Almost Always (%)**	**Always (%)**
Positive Parental Demeanor	0	0	0	30	55	15
Negative Demeanor	25	35	35	5	0	0
Consistency	0	5	30	25	25	15
Follow Through	0	0	5	35	40	20
Belittling Demeanor ^	95	5	0	0	0	0
Stern Demeanor ^	5	0	5	10	40	40

Note. Percentages were derived at the subscale level (mean items), except for single-item constructs. A Likert scale ranging from 1 (never) to 6 (always) was used to calculate percentages. ^ Single Items.

**Table 6:** Frequencies of immediate situations affecting mothers’ discipline choice (N = 20).

Immediate Situation	Very Much (%)	Often (%)	Sometimes (%)	A Little (%)	Almost Never (%)	Never (%)
Type of Misbehavior	5	10	15	20	20	30
Parent–Child Interactions	0	10	20	20	20	30
Location	0	20	15	5	40	20
Temporary Stressors	0	5	20	30	30	15

Note. Percentages were derived at the subscale level (mean items), except for single-item constructs. A Likert scale ranging from 6 (very much) to 1 (never) was used to calculate percentages.

## Data Availability

The raw data supporting the conclusions of this article will be made available by the authors upon request.

## Appendix A

**Table A1:** Discipline survey—Spanish (DS-S) item table with English translation.

Item	Spanish	English
Introductory Text	Todas las madres manejan sus problemas con el comportamiento de sus hijos en diferentes maneras. Queremos aprender sobre cómo usted ha manejado los problemas de comportamiento en los últimos tres meses con NOMBRE (NOMBRE representa el nombre de su hijo/a). Queremos que piense en lo que usted hizo cuando su hijo/a se portó mal y también como usted logro que su hijo/a se comportara de una manera beneficiosa para su futuro o que le ayudará a él/ella a llevarse bien en el mundo. Usted puede notar que algunas preguntas son similares. Esto nos ayuda a aprender con mayor precisión lo que usted hace cuando disciplina/corrige a su hijo/a.	All mothers manage their children’s behavior problems in different ways. We want to learn about how you have managed behavior problems in the last three months with NAME (NAME represents the name of your son/daughter). We want you to think more or less what you did when your child misbehaved and also how you got your child to behave in a way that would be beneficial to his/her future’ or that would help him/her to get along well in the world. You will notice that some of the questions are similar. This helps us learn with more precision what you do when you discipline your child.
Instructions	Para todas las preguntas, queremos que usted piense sobre como usted ha manejado problemas con NOMBRE en los últimos 3 meses. En los últimos tres meses cuando usted tuvo un problema con la manera en que NOMBRE se comportó, (Circule una respuesta)	For all of the questions, we want you to think about how you have managed problems with NAME in the last 3 months. In the last three months when you had a problem with the way NAME behaved, (Circle one answer)
Question Stem	En los últimos tres meses cuando usted tuvo un problema con la manera en que NOMBRE se comportó,	In the last three months when you had a problem with the way NAME behaved,
1	¿habló usted con él/ella sobre el problema?	did you talk with him/her about the problem?
Sub-question	Cuándo habló usted con NOMBRE sobre un mal comportamiento, ¿por cuánto tiempo hablan? (Marque una)	When you talked with NAME about a misbehavior, how long did you talk? (Check one)
Sub-question Responses	Yo nunca he hablado con él/ella sobre un mal comportamiento	I never talked with him/her about a misbehavior
	Menos de un minuto	Less than 1 min
	1–3 minutos	1–3 min
	3–10 minutos	3–10 min
	Más de 10 minutos, pero menos de una hora	More than 10 min but less than one hour
	Una hora o más	One hour or more
2	¿ignoró usted el comportamiento?	did you ignore the behavior?
3	¿después que le dijo que no, dejó que él/ella lo hiciera de todos modos?	after you said no, did you let him/her do it anyway?
4	en vez de disciplinar/corregir a NOMBRE, ¿dejó usted que él/ella se enfrentara a las consecuencias de su comportamiento? (Por ejemplo: ¿tener hambre cuando él/ella no quiso comer? ¿tener frío si él/ella no quiso ponerse una chaqueta? ¿tener dolor de estómago por comer demasiado dulce?)?	instead of disciplining/correcting NAME, did you let him/her deal with the consequences of his/her behavior (for example: being hungry when he/she refused to eat? being cold if he/she did not want to put on a jacket? Having a stomach-ache from eating too much candy?)?
5	¿le explicó el problema a él/ella?	did you explain the problem to him/her?
6	¿usó usted la misma disciplina, cada vez que él/ella repitió el mismo mal comportamiento?	did you use the same discipline, each time he/she repeated the same misbehavior?
7	¿le dio a NOMBRE una recompensa/premio (por ejemplo: dulce, ‘estiquer’, o ir a su lugar preferido como Sky zone o Chuck E. Cheeses) la próxima vez que él/ella enfrentó una situación similar y se portó bien?	did you give NAME a reward (for example: candy, sticker, or going to his/her favorite place like Sky Zone or Chuck E. Cheeses) the next time he/she faced a similar situation and behaved well?
8	¿le demostró a NOMBRE cómo comportarse de manera correcta cuando él/ella se portó mal para que él/ella pudiera imitar su comportamiento?	did you demonstrate to NAME how to behave in the correct way when he/she misbehaved so that he/she could imitate your behavior?
9	¿siguió usted adelante con lo que dijo que iba hacer?	did you follow through with what you said you would do?
10	¿hizo que NOMBRE se quedara solo/a por un tiempo breve (time out)?	did you make NAME stay by him/herself for a brief time (time out)?
Sub-question	Si es así, ¿por cuánto tiempo? (Marque uno)	If so, how long? Mark one
Sub-question Response Options	0–5 minutos	0–5 min
	5–10 minutos	5–10 min
	11 minutos–1 hora	11 min–1 h
	Más de una hora	More than an hour
11	¿le pego usted a él/ella? (p. ej. ‘Papao’, nalgadas, tapaboca, cocotazo)	did you hit him/her (ex. hitting on the hands, butt, mouth, or head)?
Sub-question	Por lo general, cuándo le dio (p. ej. nalgadas o ‘papao’) a NOMBRE por un mal comportamiento ¿cuántas veces le dio? (Marque una)	In general, when you hit (spank on butt or hit hands) NAME for a misbehavior, how many times did you hit him/her? (Mark one)
Sub-question Response Options	Ninguna	None
	Una	One
	Dos	Two
	Tres	Three
	Cuatro	Four
	Cinco	Five
	Más de Cinco	More than Five
12	¿sabía NOMBRE qué esperar de usted cuando él/ella se portaba mal?	did NAME know what to expect from you when he/she misbehaved?
13	¿le quitó usted los privilegios de él/ella?	did you take away his/her privileges?
Sub-question	Si es así, ¿qué le quitó? (Marque uno)	If so, what did you take away? (Mark one)
Sub-question Response Options	Juguete o juego	Toy or game
	Otro objeto que le pertenece a él/ella	Another object belonging to him/her
	Televisión	T.V.
	Vídeo o juego de vídeo	Video or video game
	Computadora/tableta	Computer/Tablet
	Comida/dulce	food/candy
	Otro	Other
Sub-question	¿Por cuánto tiempo se lo quitó? (Marque uno)	How long did you take it away? (Mark one)
Sub-question Response Options	0–5 minutos	0–5 min
	Más de 5 minutos, pero menos de una hora	More than 5 min, but less than an hour
	Más de una hora, pero menos de tres horas	More than an hour, but less than three hours
	Más de tres horas, pero menos de 12 horas	More than three hours but less than 12 h
	Más de 12 horas	More than 12 h
14	¿trató de distraer a NOMBRE para que él/ella parara el comportamiento?	did you try to distract NAME so that he/she would stop the behavior?
15	¿le dejó usted saber a NOMBRE que lo/la estaba vigilando?	did you let NAME know that you were watching him/her?
16	¿le demostró a NOMBRE buenos ejemplos de cómo comportarse en esa situación en el momento que él/ella se estaba portando mal?	in the moment he/she was misbehaving, did you demonstrate good examples for NAME of how to behave in that situation?
17	¿ya sabía usted cuál sería su disciplina cuando NOMBRE se comportará de una manera en particular?	did you already know what your discipline would be when NAME would behave in a particular way?
18	¿le dejo usted de prestar atención a NOMBRE mientras se estaba comportando de esta manera?	did you stop paying attention to NAME while he/she was behaving that way?
19	¿le dijo usted a NOMBRE por qué él/ella no debería de comportarse así?	did you tell NAME why he/she should not behave that way?
20	¿le dio en sus manos?	did you slap his/her hand?
21	¿le dejó saber a NOMBRE que usted lo/la estaba mirando para ver si el comportamiento seguía?	did you let NAME know that you were looking at him/her to see if the behavior would continue?
22	¿le mostró a NOMBRE como él/ella debería de comportarse en esa situación?	did you show NAME how he/she should behave in that situation?
23	¿actuó como si el comportamiento no estaba ocurriendo?	did you act as if the behavior was not happening?
24	¿se echó para atrás, se dio por vencida, y no disciplinó, si él/ella se ‘enfogonó’ o molesto?	did you back out, give up, and not discipline, if he/she got angry/upset?
25 *	¿le gritó a él/ella?	did you yell at him/her?
26 *	¿lo/la regañó?	did you scold him/her?
Introductory text	Ahora nos gustaría que usted piense sobre la manera en que usted disciplina a NOMBRE, y como se sintió mientras lo/la estaba disciplinando.	Now we would like you to think about the way that you discipline NAME, and how you felt while you were disciplining him/her.
Instructions	Cuando usted estaba manejando un problema de comportamiento en los últimos 3 meses, (Circule una respuesta)	When you were handling a behavior problem in the last 3 months, (Circle one answer)
27	¿diría usted que estaba calmada?	would you say you were calm?
28	¿diría que estaba enojada?	would you say you were mad?
29	¿fue respetuosa con NOMBRE?	were you respectful of NAME?
30	¿diría usted que fue mala?	would you say you were mean?
31	¿diría usted que estaba tratando de avergonzar/abochornar a NOMBRE?	would you say you were trying to embarrass NAME?
32	¿intentó que él/ella se sintiera amado/a por usted en ese momento?	did you try to make him/her feel loved by you at that time?
33	¿diría que estaba actuando de una manera firme y seria?	would you say you were acting in a firm and serious way?
34	¿le dijo cosas que lo/la motivaran?	did you say things that would motivate him/her?
35 *	¿diría que estaba frustrada?	would you say you were frustrated?
36 *	¿diría usted que se sentía abochornada o avergonzada?	would you say you were embarrassed?
37 *	¿respiró profundo varias veces antes de disciplinar a NOMBRE?	did you take some deep breaths before disciplining NAME?
38 *	¿paró y pensó cómo iba a responder antes de disciplinar a NOMBRE?	did you stop and think about how to respond before disciplining NAME?
39 *	¿reaccionó inmediatamente sin pensar cuando disciplinó a NOMBRE?	did you immediately react without thinking?
40 *	¿estaba tratando de asustar o darle miedo a NOMBRE?	would you say you were trying to scare NAME?
41	En general, ¿con qué frecuencia piensa usted que NOMBRE se porta mal? (Marque una)	In general, how often do you think NAME misbehaves? (Circle one)
Response Options ^	Nunca (1)	Never (1)
	Raramente (2)	Rarely (2)
	A Veces (3)	Sometimes (3)
	Frecuentemente (4)	Often (4)
	Casi Siempre (5)	Almost Always (5)
	Siempre (6)	Always (6)
42	¿Cuál diría usted que fue el método principal usado para disciplinarla a usted cuando era una niña (Circule una)	What would you say was the primary method used to discipline you as a child?(Circle one)
Response Options	Castigos corporales—p. ej. nalgadas, le pegaron, la empujaron	Corporal punishment-e.g., spankings, you were hit, you were pushed
	Quitando privilegios—p. ej. tiempo que usted pasaba sola como castigo, tiempo que usted no podía salir como castigo, no ver T.V.	Taking away privileges-e.g., time out (time that you would spend a lone as punishment), time that you could not go out as a punishment, no television
	Hablando sobre el problema	Talking about the problem
	Otro, por favor especifiqué	Other, please specify

Note. The Spanish version of this measure was adapted from the Discipline Survey [28] with permission. * Represents items added during pretesting of measure. ^ Represents response options for all items except for sub-questions and item 42.

**Table A2:** Immediate Situation—Spanish (IS-S) Item Table with English translation.

Item	Spanish	English
Introductory Text	Muchas cosas pueden afectar la manera en que una madre disciplina/corrige a su hijo/a. Piense en las cosas que han afectado la manera en que usted disciplina a NOMBRE en los últimos 3 meses. ¿Con cuánta frecuencia diría usted que cada una de las siguientes situaciones han afectado la manera en que usted a disciplinado a NOMBRE?	Many things can affect the way a parent disciplines his/her son/daughter. Think of the things that have affected the way that you disciplined NAME in the last 3 months. How often would you say that each of the following situations have affected the way you disciplined NAME?
Instructions	Dígame con cuánta frecuencia cada una de las siguientes situaciones han afectado la manera que usted ha disciplinado a NOMBRE en los últimos 3 meses:	Tell me how often each of the following situations affected the way you disciplined NAME in the last 3 months:
Question Stem	…¿ha afectado la manera que usted ha disciplinado a NOMBRE en los últimos 3 meses?	…has it affected the way you disciplined NAME in the last 3 months?
1	Si usted estaba teniendo un mal día	If you were having a bad day
2	Si usted estaba afuera en público	If you were out in public
3	Si alguien más estaba alrededor	If someone else was around
4	Lo que hizo NOMBRE para comportarse mal	What NAME did to misbehave
5	Si usted ya había disciplinado a NOMBRE por la misma mala conducta	If you had already disciplined NAME for the same bad conduct
6	Si usted estaba bajo mucho estrés	If you were under a lot of stress
7	La manera en que NOMBRE reaccionó hacia usted cuando usted lo/la disciplinó	The way that NAME reacted to you when you disciplined him/her
8	Si usted tenía o no tenía mucho tiempo en ese momento para bregar con la situación	If you had or did not have a lot of time at that moment to deal with the situation
9	Si usted estaba cansada	If you were tired
10	Si usted se sentía apoyada o no	If you felt supported or not
11	El número de veces que NOMBRE ya se había portado mal ese día	The number of times that NAME had already misbehaved that day
Response Options	Muchísimo (6)	Very much (6)
	Frecuentemente (5)	Often (5)
	Algunas veces (4)	Sometimes (4)
	Un Poco (3)	A Little (3)
	Casi Nunca (2)	Almost Never (2)
	Nunca (1)	Never (1)

Note. The Spanish version of this measure was adapted from the Immediate Situation measurement instrument [29], with permission.
